# Genetic effects of chemically and biosynthesized titanium dioxide nanoparticles *in vitro* and *in vivo* of female rats and their fetuses

**DOI:** 10.3389/fvets.2023.1142305

**Published:** 2023-08-08

**Authors:** Zeinab Kamal, Alaa H. Said, A. A. Ebnalwaled, Ibrahim F. Rehan, František Zigo, Zuzana Farkašová, Mohammad Allam

**Affiliations:** ^1^Department of Zoology, Faculty of Science, South Valley University, Qena, Egypt; ^2^Electronic and Nano Devises Lab, Faculty of Science, South Valley University, Qena, Egypt; ^3^Department of Husbandry and Development of Animal Wealth, Faculty of Veterinary Medicine, Menoufia University, Shebin Alkom, Egypt; ^4^Department of Pathobiochemistry, Faculty of Pharmacy, Meijo University, Nagoya, Aichi, Japan; ^5^Department of Nutrition and Animal Husbandry, University of Veterinary Medicine and Pharmacy in Košice, Košice, Slovakia; ^6^Department of Zoology, Faculty of Science, Luxor University, Luxor, Egypt

**Keywords:** *16S rRNA*, titanium dioxide, developmental toxicity, biosynthesized, particle characterization

## Abstract

With the increase in nanoparticles (NPs) products on the market, the possibility of animal and human exposure to these materials will increase. The smaller size of NPs facilitates their entrance through placental barriers and allows them to accumulate in embryonic tissue, where they can then be a source of different developmental malformations. Several toxicity studies with chemically synthesized titanium dioxide NPs (CTiO_2_ NPs) have been recently carried out; although there is insufficient data on exposure to biosynthesized titanium dioxide NPs (BTiO_2_ NPs) during pregnancy, the study aimed to evaluate the ability of an eco-friendly biosynthesis technique using garlic extract against maternal and fetal genotoxicities, which could result from repeated exposure to TiO_2_ NPs during gestation days (GD) 6–19. A total of fifty pregnant rats were divided into five groups (n = 10) and gavaged CTiO_2_ NPs and BTiO_2_ NPs at 100 and 300 mg/kg/day concentrations. Pregnant rats on GD 20 were anesthetized, uterine horns were removed, and then embryotoxicity was performed. The kidneys of the mothers and fetuses in each group were collected and then maintained in a frozen condition. Our results showed that garlic extract can be used as a reducing agent for the formation of TiO_2_ NPs. Moreover, BTiO_2_ NPs showed less toxic potential than CTiO_2_ NPs in HepG_2_ cells. Both chemically and biosynthesized TiO_2_ NP-induced genetic variation in the *16S rRNA* sequences of mother groups compared to the control group. In conclusion, the genetic effects of the *16S rRNA* sequence induced by chemically synthesized TiO_2_ NPs were greater than those of biosynthesized TiO_2_ NPs. However, there were no differences between the control group and the embryo-treated groups with chemically and biologically synthesized TiO_2_ NPs.

## Graphical Abstract

Schematic carton of the study showed the experimental strategy. It was designed by BioRender.com. 
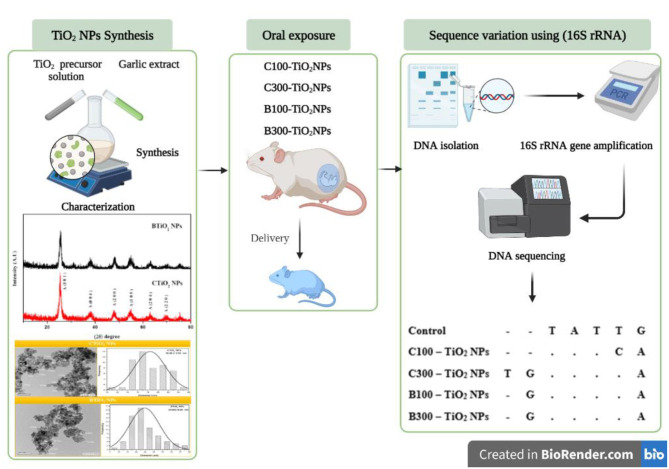

## Introduction

In the coming decades, industrial production is expected to change as a result of the rapidly expanding field of nanotechnology. The high surface area and smaller particle size of NPs, along with their flexible design, functionalization, biocompatibility, and bioactivity, played a key role in tuning NPs in many applications. The major application is breaking the barriers among fundamental fields such as biology, chemistry, and physics. The application field is also expanding, including medical products, imaging techniques, sporting equipment, food production and agriculture, cosmetics, clothing cleaning products, personal care items, and toys for kids (1, 2). However, most nanomaterials have been introduced into the market based on claimed advantages, and the ecotoxicological potential of nanomaterial products is unclear to the scientific community (2, 3). The nanomaterials' physicochemical properties are due to their chemical nature, small size, surface composition, and aggregation (4).

For NP synthesis, there are two main approaches: the top-down method and the bottom-up method (5). The top-down approach starts with size reduction from the bulk material to the nanosized with physical methods such as ball milling and laser ablation (6), while the bottom-up approach needs a reducing agent to decrease the size of the particle. Chemical (for example, sodium hydroxide and potassium hydroxide) and biological [biomolecules from bacteria (6), fungi (7), yeast (8), virus (9), and plant extracts (10)] reducing agents can work perfectly for controlling the size of metals and metal oxides. Biomolecules are preferred due to their safety and biocompatibility with DNA, proteins, enzymes, polyphenols, flavonoids, and sugars (11). The simplest and quickest way to create NPs, which are based on the proteins and carbohydrates in biomolecules and act as a reducing agent to encourage the synthesis of metallic nanoparticles, is through plant extracts (12). Biomolecules have many chemically active groups such as hydroxyl, amine, and thiol groups (13). These groups will interact with metal ions in the solution via electron transfer, leading to oxidization from a positive oxidization state to a zero oxidization state, which guides the nucleation process.

Comparing biosynthesized NPs with those made using chemical methods, the biocompatibility of these NPs allows for a wide range of biomedical applications (14).

Exposure to TiO_2_ NPs may change the cell membrane due to oxidative stress or even *via* Van der Waal forces with the cell wall, leading to defragmentation of the cell membrane and molecular structure, which induces genetic variation (15, 16). The generated reactive oxygen species (ROS) also oxidize DNA, resulting in mutations in DNA (15, 17). In addition, reactive oxygen species may promote inflammation, and oxidative stress and inflammation result in cell apoptosis (16, 18).

Recently, several toxicity studies with chemically synthesized titanium dioxide nanoparticles (CTiO_2_ NPs) have been performed. Organs such as the kidney, liver, and spleen can suffer damage due to exposure to TiO_2_ NPs (19–21), such as inflammation of renal tissue due to ROS. In addition, TiO_2_ NPs accumulate in the kidney tissues and cause changes in embryogenesis during the first trimester of pregnancy (22, 23). In addition, oxidative stress significantly increases with increasing TiO_2_ NP concentrations (24).

A repeated oral administration of CTiO_2_ NPs in other experimental animals showed disturbances in metabolism (25). The results of the studies performed on rats as animal models revealed that after absorption of CTiO_2_ NPs can enter the systemic circulation and cause organ injuries and inflammation (26).

Several *in vivo* and *in vitro* studies were carried out to assess the toxicity of green TiO_2_ NPs. The reported toxicological data proposed that green TiO_2_ promotes a higher safety profile with improved anticancer, antibacterial, and antiviral activities compared with chemically synthesized TiO_2_ NPs (27). However, there are limited toxicological data on exposure to biosynthesized TiO_2_ NPs from garlic extract (BTiO_2_ NPs) during pregnancy. This study is based on the new method of biosynthesis of TiO_2_ NPs by using garlic extract as a reducing agent, performing *in vivo* investigations on pregnant rats and their fetuses, and comparing the toxicity of BTiO_2_ NP synthesis with that of CTiO_2_ NP synthesis in both *in vivo* and *in vitro*.

## Materials and methods

### Chemicals and reagents

The following chemicals and reagents were used without any purification: titanium isopropoxide (TTIP), Dulbecco's modified Eagle's medium (DMEM), and penicillin–streptomycin (P/S) were purchased from Sigma–Aldrich Co., USA. Fetal bovine serum (FBS) was purchased from Biochrom, and 0.05% Trypsin-EDTA was purchased from HiMedia, India. Phosphate-buffered saline (PBS) was purchased from Serox, Germany, and 3-(4,5-dimethylthiazol-2-yl)-2, 5-diphenyltetrazolium and DMSO were purchased from Lobalohemia, India. Qiagen DNA mini kit and 100 bp DNA Ladder were purchased from GeneDireX (Germany). Liver hepatocellular cells (HepG_2_ cell lines) were purchased from VACSERA, Giza, Egypt.

### Synthesis of chemical TiO_2_ NPs (CTiO_2_ NPs)

TiO_2_ NPs were created chemically using the coprecipitation method (28). In brief, 5 ml of titanium isopropoxide (TTIP) in 15 ml of propanol was used as a precursor solution, while the solvent solution was a 50/1 (V/V) mixture of distilled water and propanol. The precursor solution was added dropwise to the solvent solution after it had been heated to 70–90°C under continuous stirring for 2 h. As soon as the TiO_2_ precursor was reduced to form TiO_2_ NPs, a white precipitate started to form. The precipitate was centrifuged and allowed to cool at room temperature for the entire next day. The final precipitate was, then, dried at 100°C for 12 h and calcined at 400°C for 3 h after being washed three times with distilled water and once with ethanol.

### Biosynthesis of TiO_2_ NPs using garlic extract (BTiO_2_ NPs)

Garlic (*Allium sativum*) water extract was used as a reducing agent for the biosynthesis of TiO_2_ NPs. Approximately 20 g of washed and dried garlic in 150 ml of distilled water were boiled for 1 h to prepare the solvent solution. The precursor solution was prepared by adding 10 ml of TTIP to 150 ml of distilled water and vigorously stirring. Dropwise addition of a solvent solution (60 ml) of fresh garlic plant extract was made, while the precursor solution was continuously stirred for 2 h. The color of the solution changed from white to dark yellow, indicating that TTIP was reduced and BTiO_2_ NPs were created. The formed precipitate was centrifuged and collected after being allowed to cool at room temperature for the entire night. The final precipitate was, then, dried at 100°C for 12 h and calcined at 400°C for 3 h after being washed three times with distilled water and once with ethanol.

### TiO_2_ NP characterizations

The produced TiO_2_ NPs were categorized by X-ray diffraction (XRD) using X' Pert PRO-PAN, Malvern Panalytical, UK, diffractometer with cooper radiation (wavelength 1.54056 Å) at 40 kV and 30mA, high-resolution transmission electron microscopy (HETM) (JEOL, JEM 2100, Japan), Raman spectrometer (Horiba Jobin Yvon HR 800UV, Japan), and FTIR spectrophotometer (Model 6100, Jasco-Japan), with a resolution of 4.00 cm^−1^and covers the wave number range of 4000–400 cm^−1^ was used to determine the functional groups in the prepared samples. The optical absorption spectra of the prepared samples were evaluated with a UV–visible spectrophotometer (SPECORD 200 PLUS, Analytik Jena, Germany).

### Cell viability

Liver hepatocellular cells (HepG_2_ cell lines) were obtained from Vacsera (Giza, Egypt). Cells were cultured in Dulbecco's modified Eagle's medium (DMEM, Sigma–Aldrich, #D5796), supplemented with 10% heat-inactivated fetal bovine serum (FBS, Biochrom, #S0615) and 1% penicillin–streptomycin (P/S, Sigma–Aldrich, #P4333). Cells were grown in 75 cm^2^ flasks (VACSERA Center, Cairo) and sub-cultured at approximately 80% confluency.

MTT assay was used to assess the mitochondrial function by reducing the tetrazolium dye MTT 3-(4, 5-dimethylthiazol-2-yl)-2, 5-diphenyltetrazolium to the insoluble magenta formazan where a yellow tetrazolium is reduced to purple formazan in living cells (29). HepG_2_ cells were seeded in 96-well culture plates with a total density of 1 × 10^4^ cells per well and incubated at 37°C and 5% CO_2_. After 24 h of incubation, the culture medium was removed, and cells were rinsed with 100 μl PBS. Afterward, cells were exposed to both samples of TiO_2_ NPs with different concentrations (0, 0.5, 1, 2, 4, and 8 mM) and then incubated at 37°C and 5% CO_2_. After 24 h, cells were rinsed with PBS, and 80 μl of media without serum and 20 μl of MTT solution were added to each well and then incubated at 37°C for 3 h. Finally, the MTT solution was removed, then 100 μl of MTT solvent (DMSO) was added to each well, and the plates were warped in foil and shaken on an orbital shaker for 15 min. The absorbance at OD = 590 nm was recorded for each well with a Tecan infinite F50 absorbance microplate reader, and the cell viability was calculated using the following equation (29).


$$\text{Cell viability}\%\text{ =}\frac{{\text{Absorbance}}_{\text{control}}{-\text{Absorbance}}_{\text{sample}}}{{\text{Absorbance}}_{\text{control}}}$$

### Animal experiment

In total, 20–30-week-old healthy female Albino Sprague–Dawley rats, weighing 180–220 g, were obtained from the animal house of the National Research Center Institute (Cairo, Egypt). The rats were housed in cages made of plastic, using a bedding material made of a wooden dust-free litter, and permitted a 2-week period of acclimation before the beginning of the study under hygienic measures and standard conditions (25 ± 2°C room temperature, 50% ± 15% relative humidity, and light–dark cycle of 12 h). All rats were given a commercial pellet diet and water *ad libitum*.

### Experimental design and treatment

The study aimed to assess the ameliorator abilities of an eco-friendly biosynthesizing method using garlic (*Allium sativum*) extract as a reducing agent against maternal and fetal genotoxicities that could develop from repeated oral administration of TiO_2_ NPs in pregnant mothers between gestation days 6 and 19. In total, 50 adult female rats were selected for this study and mated at night using healthy males in a ratio of 1:3. The day that sperm was observed in the vaginal smear, the next morning was regarded as GD1. After pregnancy detection, pregnant females were weighed and assigned into five equal groups, each of which comprises 10 pregnant mothers, as follows:

*Group 1*: control group, administrated distilled water by gavage (1 ml/day).

*Group 2* and *Group 3*: CTiO_2_ NP-treated groups received 100 and 300 mg/kg body weight (bw)/day, (30, 31) respectively.

*Group 4* and *Group 5*: BTiO_2_ NP-treated groups received 100 and 300 mg/kg bw/day, respectively. These concentrations of TiO_2_ NPs of 100 and 300 mg/kg bw were chosen according to the World Health Organization (WHO) in 1969 (32), which reported that the LD_50_ of TiO_2_ for rats is greater than 12,000 mg/kg bw. Pregnant mothers were exposed to 100 mg/kg bw CTiO_2_ NPs or BTiO_2_ NPs, which is equal to approximately 6–7 g of TiO_2_ NPs per 60–70 kg bw for humans (33).

TiO_2_ NPs were suspended in distilled water. Throughout the dosing process, the dosage was continuously shaken to produce a homogenized suspension, and throughout the dosing procedure, the dosage was continuously stirred with a magnetic stirrer. All treatments were obtained by oral gavage and were freshly prepared. The pregnant rats' treatment starts from GD 6 to GD 19 (31). The schematic diagram of the study showed the experimental strategy (see Graphical abstract).

At the end of the experimental period on GD 20, all dams were euthanized by ether, and cesarean sections were performed. The uterine horns were removed, and then, the embryotoxicity was performed. The kidneys of mothers and fetuses in each group were collected and then maintained in a frozen condition.

### DNA extraction, PCR amplification, and sequencing

Qiagen DNA mini kit was used to extract the genomic DNA from the kidneys of mothers and fetuses in each group by following the manufacturer's guidelines. The quality of DNA was examined by agarose gel electrophoresis with a 100bp DNA Ladder under UV light. The previous report stated that primers were used for *16S rRNA* amplification (34). The polymerase chain reaction was executed in a final reaction volume of 40 μl, adding 20 μl of 2X Master Mix, 1 μl of each forward and reverse primer, 17 μl of nuclease-free water, and 1 μl genomic DNA. The PCR conditions were as follows: denaturing at 95°C for 4 min, then 35 cycles of denaturing at 94°C for 60 s, annealing at 48°C for 60 s, extension at 72°C for 60 s, and finishing with an extension at 72°C for 7 min. Amplification was confirmed by means of agarose gel electrophoresis at 1.5% containing ethidium bromide. The DNA sequencing was executed by Macrogen (South Korea). Sequence alignment was implemented using Clustal W (35).

### Biopsy of fetal kidney

Fetal kidney biopsies from all embryos were extracted and thoroughly dipped in formalin solution to be fixative, followed by serial dehydration in ethanol, and embedding in paraffin wax. Kidney sections of approximately 5 microns were stained with hematoxylin and eosin (H&E) for histological findings (36).

## Results

### X-ray diffraction

**Figure 1:**
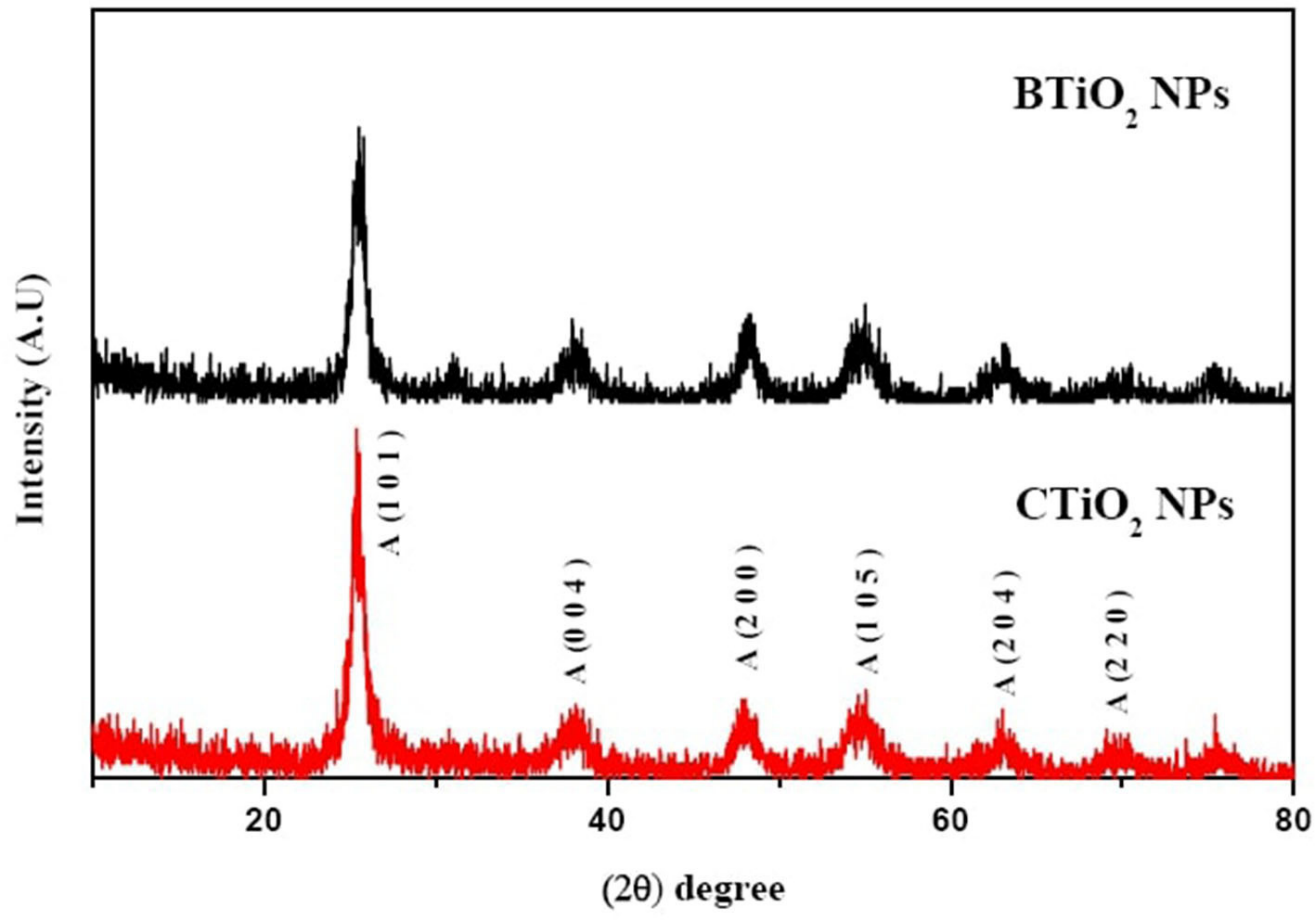
XRD patterns of CTiO_2_ and BTiO_2_ NPs.

The biosynthesis of NPs is an emerging technique that produces NPs with unique properties. Green nanotechnology was applied to the synthesis of TiO_2_ NPs, and the formed NPs were characterized by several techniques. Structural analysis using XRD, HRTEM, and Raman spectroscopy revealed anatase phase formation, which indicates the complete reduction of titanium isopropoxide using garlic extract. No additional peaks for impurities, such as NaCl and Na_2_TiO_3_, were observed, indicating the high purity of the prepared samples. The X-ray diffraction (XRD) pattern of TiO_2_ NPs (see Figure 1) revealed the phase and structural purity of TiO_2_ NPs. All the recorded peaks belong to the anatase phase TiO_2_ NPs, which support the reported card (JCPDS No. 21-1272). Although the diffraction peak of brookite B (*121*) was found in CTiO_2_ NPs, it disappeared in BTiO_2_ NPs (37).

Debye–Scherer's equation was used for the calculation of the average crystal size of TiO_2_ NPs (38).


$$\begin{array}{l} d=\frac{0.89\lambda }{FWHM\;Cos\theta } \end{array}$$

where *d* is the average crystal size of TiO_2_ NPs, λ is the wavelength of X-rays, 0.89 is a constant, θ is the diffraction angle, and FWHM is the full width at half maximum of XRD peaks recorded at diffraction angle 2θ. The calculated average crystalline size of CTiO_2_ NPs was 48.11 nm, while it was increased to 53.31 nm for BTiO_2_ NPs.

### UV–visible spectroscopy

**Figure 2:**
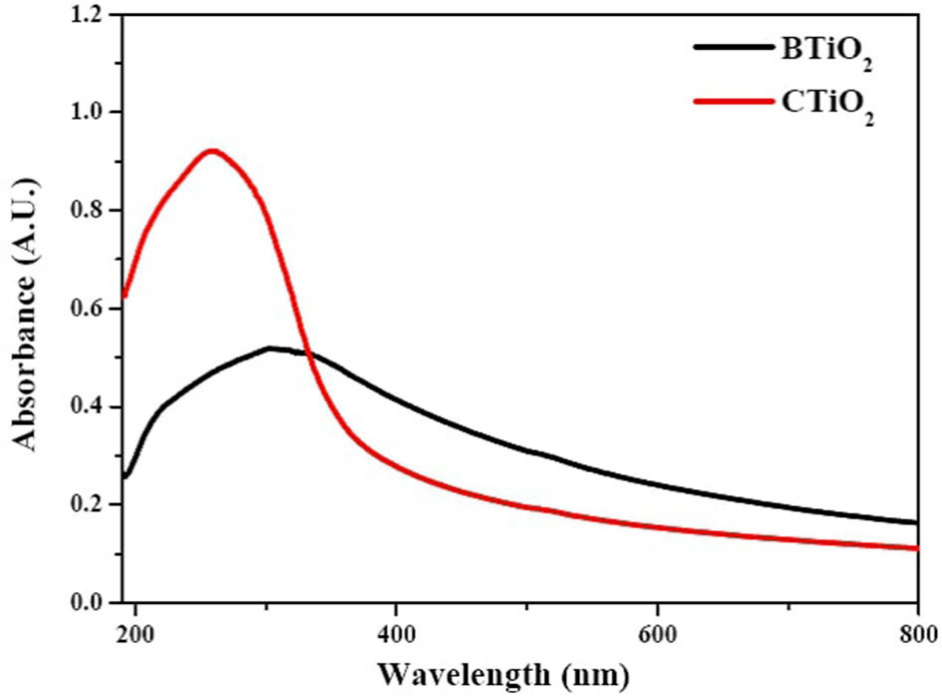
UV spectra of BTiO_2_ and CTiO_2_ NPs.

Both CTiO_2_ and BTiO_2_ samples have absorption spectra in the region below 400 nm. The spectral image displays the absorption peaks of TiO_2_ NPs at wavelengths of 261.87 and 314 nm for CTiO_2_ and BTiO_2_ NPs, respectively (see Figure 2).

The optical band gap Eg of TiO_2_ NPs was calculated by Tauc's equation (39) as follows:


$$\begin{array}{l} \propto \left(hv\right)={A\left(hv-E_{g}\right)}^{\frac{m}{2}} \end{array}$$

where **∝** is the absorption coefficient of TiO_2_ NPs, the energy of incident light of wavelength λ was *hv* = hc/λ, (A) was constant, and (*m*) depends on the nature of the transition.

**Figure 3:**
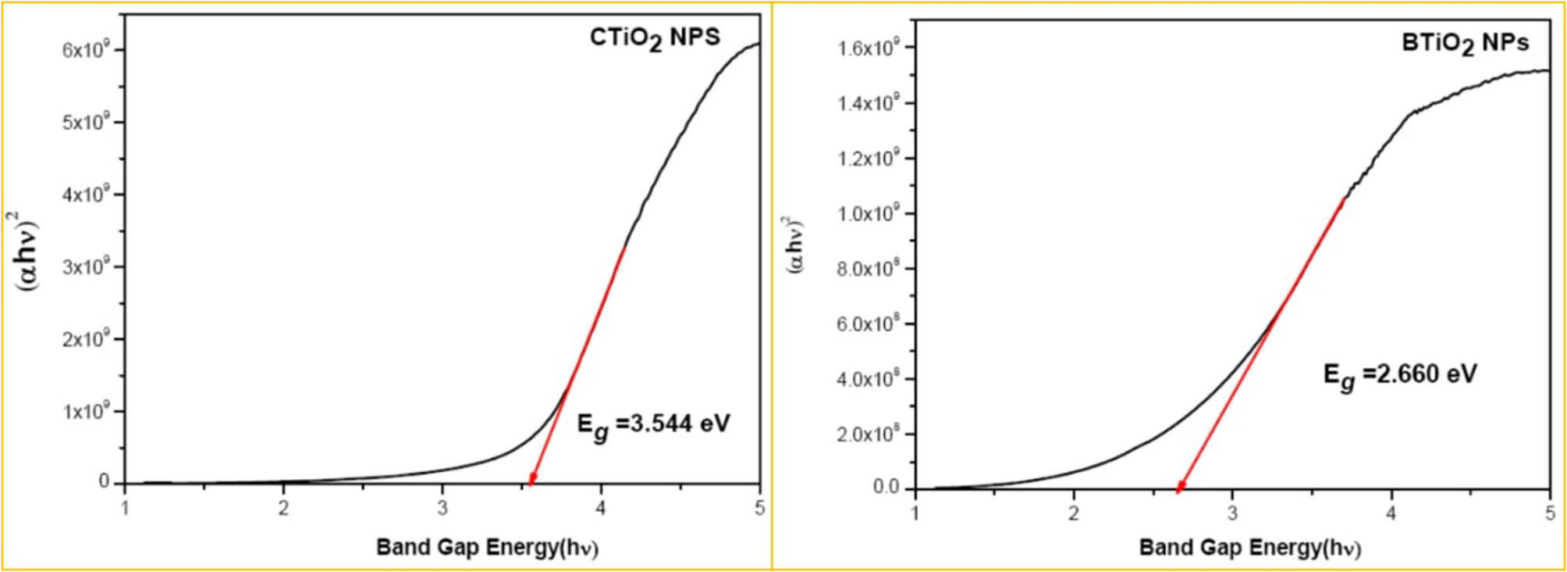
Optical band gap of CTiO_2_ and BTiO_2_ NPs.

The direct band gaps of CTiO_2_ and BTiO_2_ NPs were 3.544ev and 2.660ev, respectively (see Figure 3).

### Raman spectra analysis of tio_2_ NPs

The structural characterization of chemically and biosynthesized TiO_2_ NPs with garlic extract by Raman spectroscopy is presented in Figure 3. The six active modes that belong to the anatase phase of TiO_2_ were found in the two samples, which confirm the XRD result of the formation of anatase TiO_2_ NPs. The low frequency O-Ti-O bending oscillation was observed for all samples as E_g(1)_, E_g(2)_, and B_1g_ modes (40). All sample frequency positions are higher than the reported values for bulk TiO_2_ (143, 197, and 399 cm^−1^, respectively). Moreover, higher frequency Ti-O strain oscillation was observed for all samples in E_g(3)_ and A_1g_ modes. The frequency position of E_g(3)_ has a lower frequency position than that of the bulk TiO_2_ (639 cm^−1^), while the frequency position of A_1g_ has a higher frequency position than that of the bulk TiO_2_ (514 cm^−1^). This result is attributed to a reduction in the crystal size of TiO_2_ NPs that are produced chemically and biologically (41).

### Fourier-transform infrared spectra

**Figure 4:**
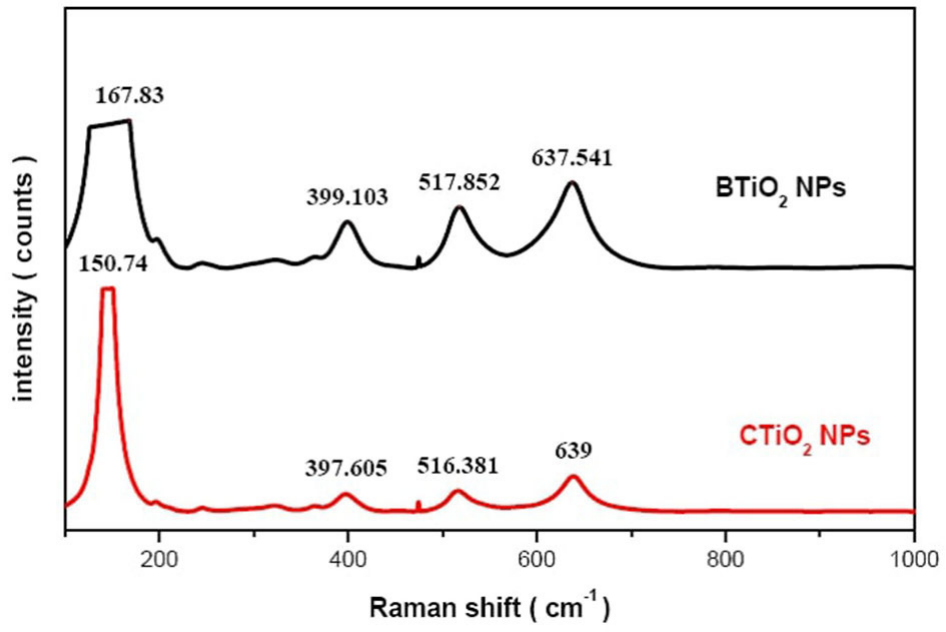
Raman spectra of CTiO_2_ and BTiO_2_ NPs.

FTIR spectrum for CTiO_2_ NPs is presented in Figure 4. The broad band at 3426.2 cm^−1^ is assigned to the vibration of the hydroxyl group (42). The vibration band at 1151cm^−1^ is due to Ti-O-Ti vibrations. The band at 1628 cm^−1^ is described as the bending vibration of adsorbed water and Ti-OH (43). A band at 1376 cm^−1^ is attributed to the asymmetrical C–H vibrations (43, 44). The band at 457.6 cm^−1^ corresponds to the Ti–O –Ti. Vibrational bands appearing around 723 cm^−1^ correspond to TiO_2_ modes in the anatase phase (45–47).

FTIR spectrum of garlic extract was studied to identify the presence of garlic function groups. The band at 3415.5cm^−1^ describes the vibration of the hydroxyl group (O-H) and the presence of carbohydrates and amino acids. The bands at 2932 cm^−1^ and 2890.2 cm^−1^ represent the C-H stretching for lipids (48). Band 1631 cm^−1^ represents the Amid I: C=O stretching for proteins. Band 1412.5cm^−1^ represents the CH_2_ vibration for lipids. The band at 1378.8 cm^−1^ represents the C=S stretching for sulfur compounds. The band at 1274.4 cm^−1^ represents the C-N stretching for amino acids. The band at 1120.5 cm^−1^ represents the C-N stretching for amino acids, symmetric C-H stretching, and the vibration presence of antioxidant enzymes. The band at 1025.6 cm^−1^ represents SO_3_ symmetric stretching, mainly sulfur compounds. Bands at 931.2 cm^−1^ and 817.5 cm^−1^ represent the N-H stretching of proteins (49).

FTIR spectroscopy indicated the successful formation of TiO_2_ NPs and the generation of the functional groups in garlic extract that are responsible for the reduction of Ti precursors. The FTIR spectrum of TiO_2_ NPs is characterized by three broad bands, the first of which, between ~3800 and 3000 cm^−1^, belongs to the stretching vibration of the hydroxyl group (O-H). The second band is around 1626 and 1638 cm^1^, which belongs to the stretching of titanium. The third band, located approximately between 800 and 450 cm^−1^, was the fingerprint of Ti-O stretching vibration. These bands were observed in all samples, which confirms the formation of TiO_2_ NPs (50).

The biosynthesis of TiO_2_ NPs using garlic extract was confirmed by the occurrence of functional groups in both TiO_2_ and garlic. The functional groups of garlic at 1631 cm^−1^, 1378.8 cm^−1^, and 1120.5 cm^−1^ were shifted to the lower wave numbers such as 1623.6 cm^−1^, 1377 cm^−1^, and 1117.4 cm^−1^, respectively. This reflects the presence of C=O, C=S, and C-N and symmetric C-H stretching vibrations of antioxidant enzymes in the formation of TiO_2_-garlic NPs. The band at 417.7 cm^−1^ corresponds to the Ti–O –Ti. A vibrational band appeared around 716.8 cm^−1^ due to TiO_2_ modes in the anatase phase (51). The bands assigned for C-H stretching, CH_2_ vibration, and symmetric C-H stretching disappeared in biosynthesized TiO_2_NPs. In addition, bands corresponding to C=O, C=S, and C-N appeared in biosynthetic TiO_2_ NPs. Consequently, C=O, C=S, and C-N functional groups are the cause of the bioreduction of titanium isopropoxide to TiO_2_NPs.

### High-resolution transmission electron microscope

**Figure 5:**
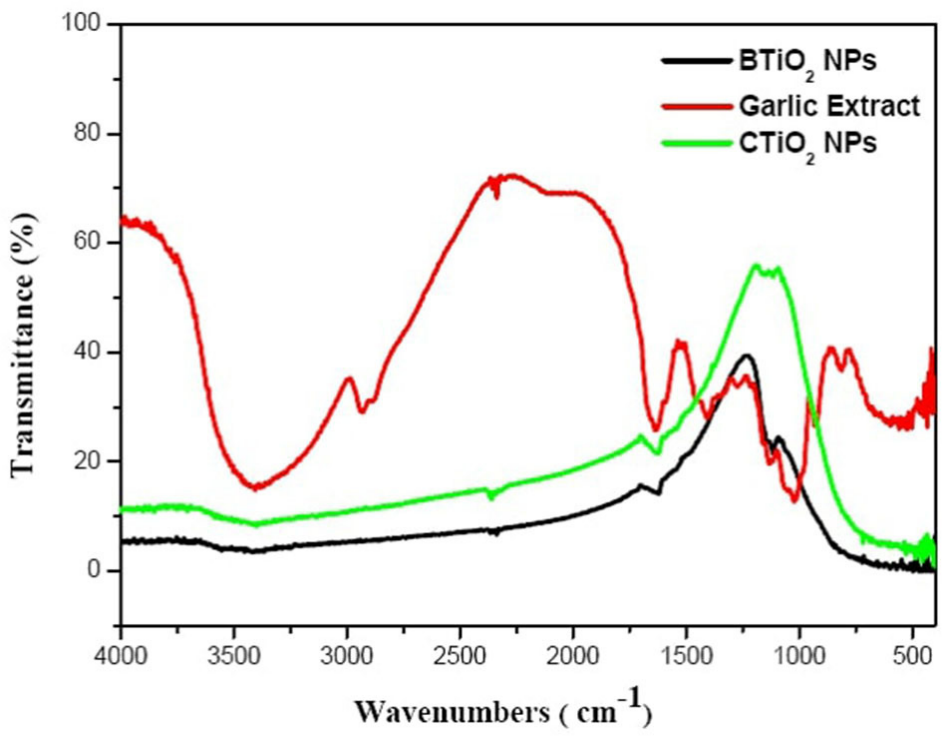
FTIR spectra of garlic extract and BTiO_2_ and CTiO_2_ NPs.

HRTEM images of CTiO_2_ and BTiO_2_ NPs reflect a sort of agglomeration with a spherical or irregular spherical shape (see Figure 5). Particle size distribution of CTiO_2_ and BTiO_2_ NPs (see Figure 5) is estimated from HRTEM images. HRTEM images of the prepared samples showed a sort of agglomeration with an increase in the particle size of BTiO_2_ NPs compared to CTiO_2_ NPs, which was attributed to the presence of the biomolecules as they attracted other molecules due to the electrostatic force on their surfaces. Similar results were also obtained in the previous report (52, 53).

The average size is 18.46 ± 3.03 and 35.64 ± 4.9 nm for CTiO_2_ and BTiO_2_ NPs, respectively. These values are smaller than the calculated values from XRD results (48.19 and 53.317) for CTiO_2_ and BTiO_2_ NPs, respectively. This variation in size may be due to the agglomeration of TiO_2_ NPs (51). The crystallite size calculated from Scherer's equation is the apparent size, which does not equal to the particle size, especially in the case of polydisperse NPs with aggregation, such as TiO_2_ NPs.

### Cytotoxicity

**Figure 6:**
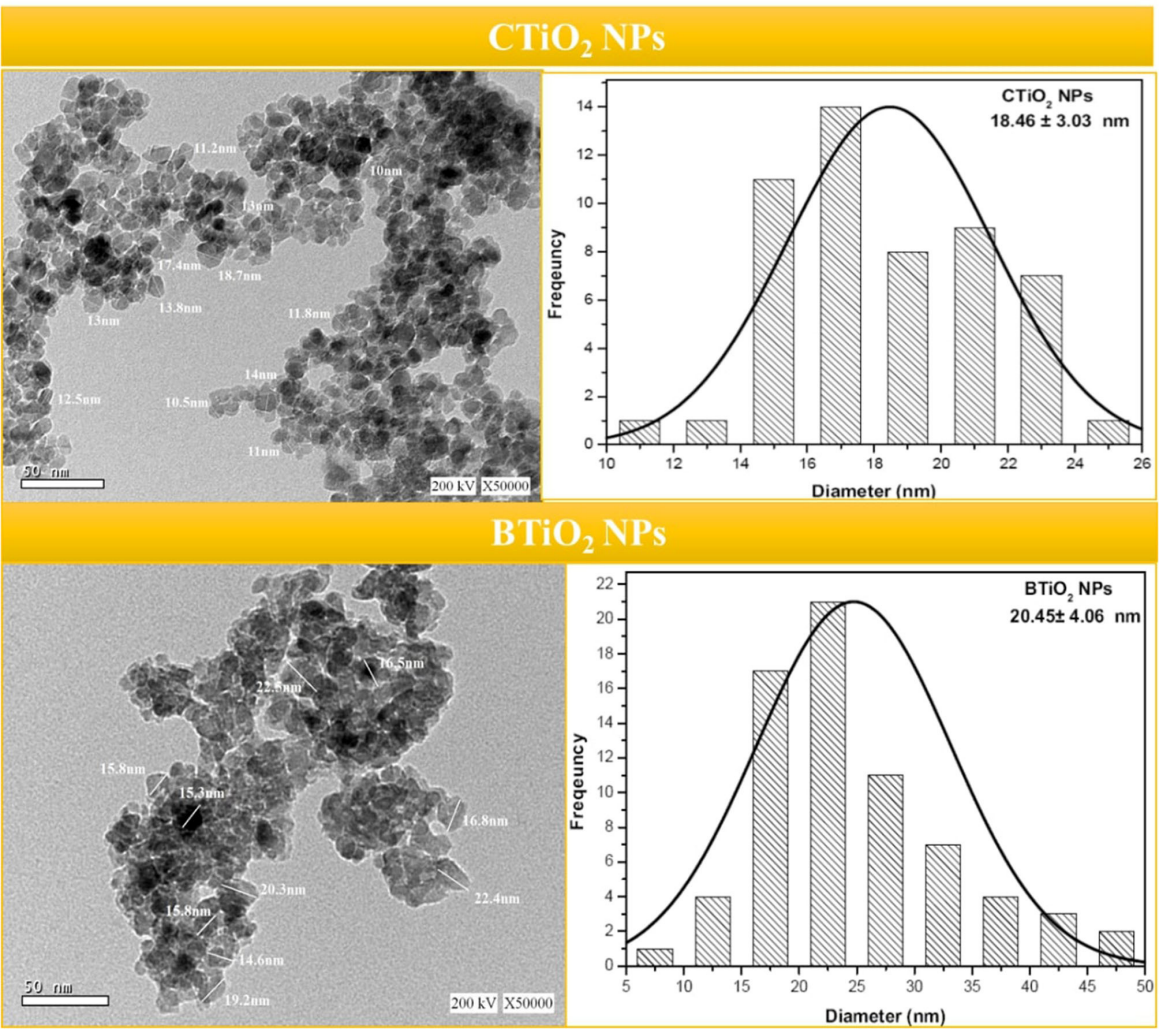
HRTEM images and particle size distribution of BTiO_2_ and CTiO_2_ NPs.

**Figure 7:**
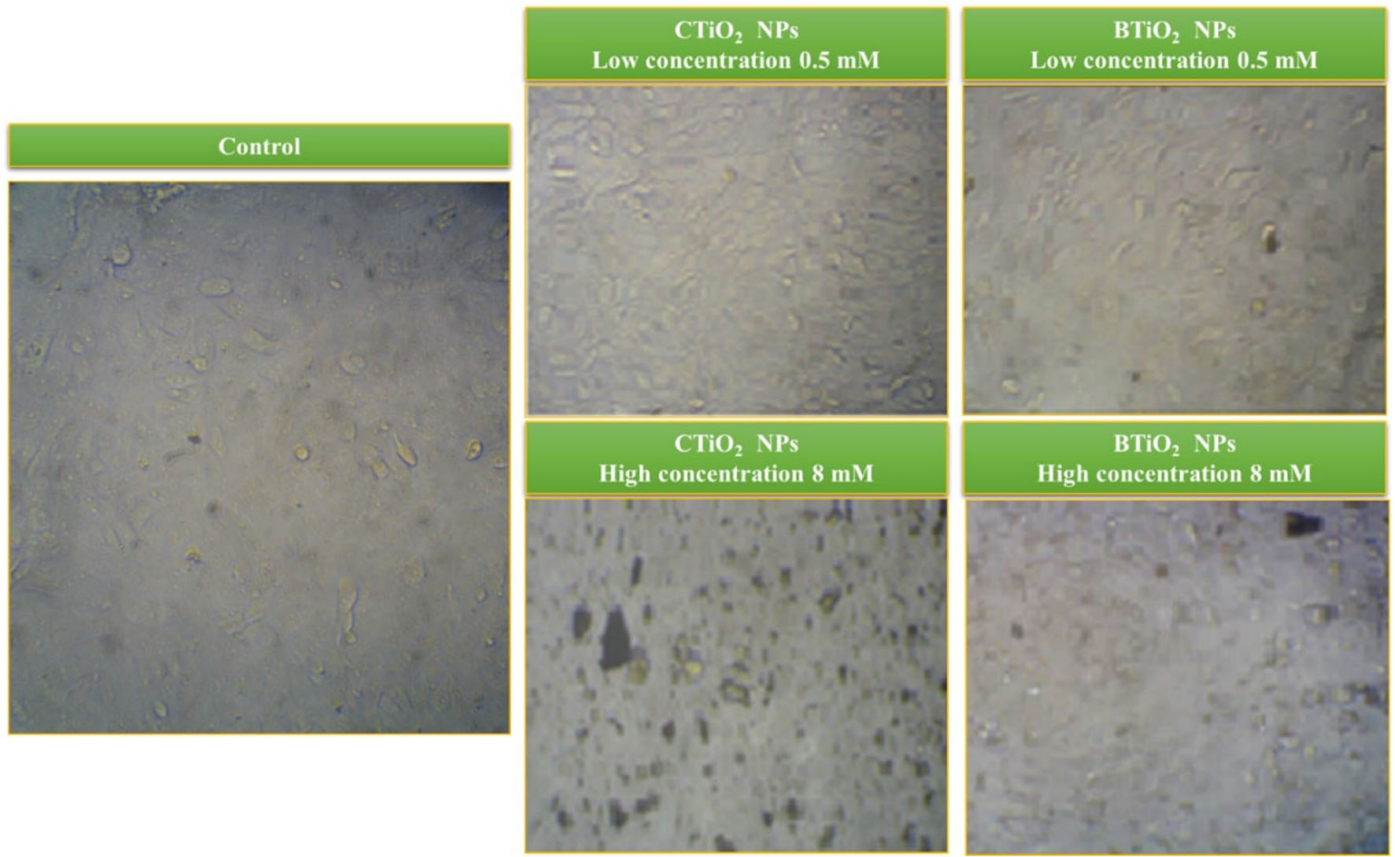
Microscopic images of HepG_2_ cells after 24 h of exposure to BTiO_2_ and CTiO_2_ NPs at two concentrations (0.5 and 8 mM).

**Figure 8:**
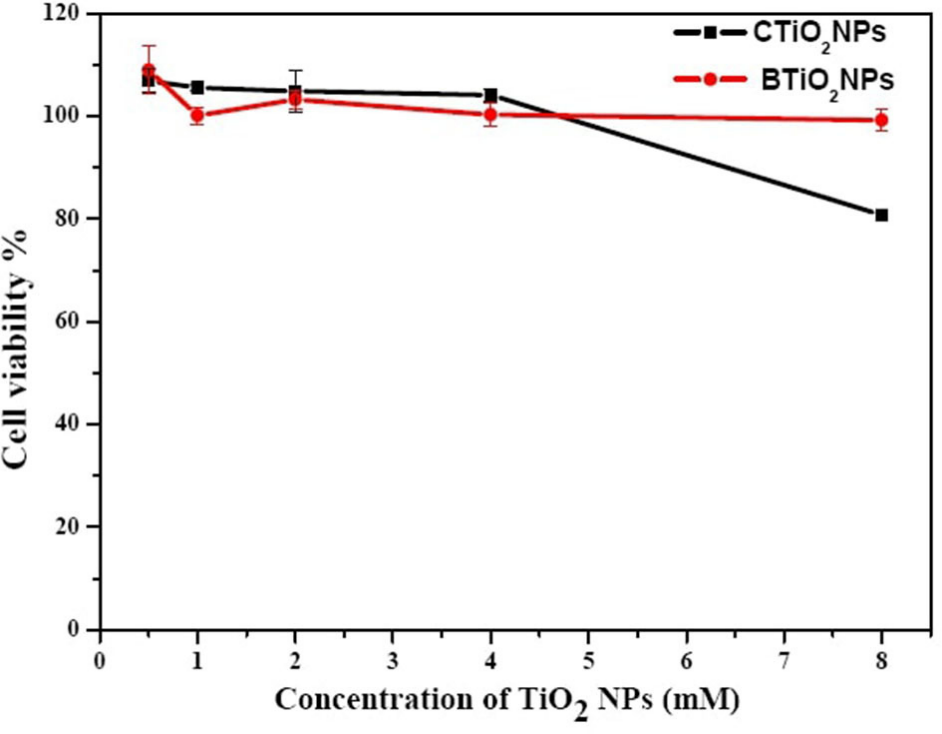
Cell viability of HepG_2_ cells after 24 h of exposure to BTiO_2_ and CTiO_2_ NPs at two concentrations (0.5 and 8 mM).

An accumulation of both TiO_2_ NPs on the surface of the HepG_2_ cells was found in the microscopic images (see Figure 6). Particularly, in the case of CTiO_2_ NPs, this accumulation increased as NP concentration increased. The normalized cell viability is presented in Figure 7, and there is no observed toxicity for CTiO_2_ and BTiO_2_ NPs at low concentrations, while a small percentage of toxicity was observed for CTiO_2_ at high concentrations (8 mM), as shown in Figure 8.

The cytotoxicity of biosynthesized NPs can be tuned by several parameters, such as particle size, shape, and surface chemistry (54). It is reported that the biosynthesis of NPs can modify their surface due to the interaction with biomolecules, which, in turn, enhances the biocompatibility of the formed NPs (55). Similar behavior was observed in the cytotoxicity of our samples, as BTiO_2_ NPs showed less toxic potential than CTiO_2_ NPs. The accumulation of TiO_2_ NPs on the surface of HepG_2_ cells reduces the internalization rate and the cytotoxic effects (56–58).

### Sequence variation using *16s rRNA* gene

Herein, we assess the potential genetic effects of oral exposure to chemically and biosynthesized TiO_2_ NPs with two doses (100 and 300 mg/kg body weight/day) during pregnancy.

In mothers, 538–543 bp of nucleotide sequences were obtained. The obtained sequences were deposited into GenBank, and the accession numbers are MZ782915, MZ782917, MZ782918, MZ782919, and MZ782920. The percentages of nitrogen bases are shown in Table 1. In the embryo, 540–550 bp of nucleotide sequences were obtained. The obtained sequences of embryos were submitted to GenBank, and the accession numbers are MZ788644, MZ788646, MZ788647, MZ788648, and MZ788649. The percentages of nitrogen bases are shown in Table 2.

**Table 1:** Accession numbers, nucleotide frequencies, and their averages for *16S rRNA* gene in the five mother groups.

**Group**	**Accession number**	**Base pair Length**	**Nucleotide Number%**				**A + T Content (%)**	**C + G Content (%)**
			**A%**	**T%**	**C%**	**G%**		
Control	MZ782915	538.0	33.3	27.3	19.7	19.7	60.6	39.4
C100 – TiO_2_ NPs	MZ782917	539.0	33.4	27.2	19.9	19.5	60.6	39.4
C300 – TiO_2_ NPs	MZ782918	543.0	33.1	27.7	19.5	19.7	60.8	39.2
B100 – TiO_2_ NPs	MZ782919	542.0	33.2	27.3	19.6	19.9	60.5	39.5
B300 – TiO_2_ NPs	MZ782920	542.0	33.2	27.3	19.6	19.9	60.5	39.5
Average%		540.8	33.2	27.5	19.6	19.7	60.7	39.3

**Table 2:** Accession numbers, nucleotide frequencies, and their averages for *16S rRNA* gene in the embryo in five groups.

**Group**	**Accession number**	**Base pair Length**	**Nucleotide number%**				**A + T Content (%)**	**C + G Content (%)**
			**A%**	**T%**	**C%**	**G%**		
Control	MZ788644	540.0	33.4	27.4	19.6	19.6	60.8	39.2
C100 – TiO_2_ NPs	MZ788646	540.0	33.4	27.4	19.6	19.6	60.8	39.2
C300 – TiO_2_ NPs	MZ788647	546.0	33.2	28	19.4	19.4	61.2	38.8
B100 – TiO_2_ NPs	MZ788648	550.0	33	28.2	19.3	19.5	61.2	38.8
B300 – TiO_2_ NPs	MZ788649	550.0	33.3	28	19.3	19.4	61.3	38.7
Average%		544.3	33.3	27.7	19.5	19.5	61	39

**Figure 9:**
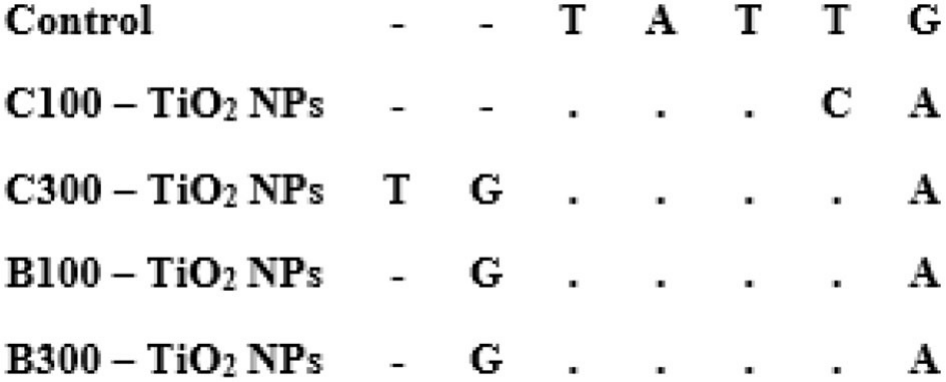
Alignment of *16S rRNA* partial sequences in mother-five groups. Dots refer to identical nucleotides, and A, T, C, and G refer to different nucleotides.

In mothers, the P-distance among the groups was 0.0000 to 0.0027%. The highest P-distance (0.0027) was found between the control group and C100–TiO_2_ NPs groups (Table 3 and Figure 9). In the embryo, the P-distance among the groups was 0.00. Overall, the distance value among all groups was 0.000% (Table 4).

**Table 3:** Pairwise distances among mother-five groups using *16S rRNA* gene.

**Control**		0.0027	0.0018	0.0018	0.0018
**C100 – TiO**_2_ **NPs**	0.0037		0.0020	0.0020	0.0020
**C300 – TiO**_2_ **NPs**	0.0019	0.0019		0.0019	0.0019
**B100 – TiO**_2_ **NPs**	0.0019	0.0019	0.0018		0.0000
**B300 – TiO**_2_ **NPs**	0.0019	0.0019	0.0018	0.0000	

**Table 4:** Pairwise distances among embryonic five groups using *16S rRNA* gene.

**Control**		0.000	0.000	0.000	0.000
**C100 – TiO**_2_ **NPs**	0.000		0.000	0.000	0.000
**C300 – TiO**_2_ **NPs**	0.000	0.000		0.000	0.000
**B100 – TiO**_2_ **NPs**	0.000	0.000	0.000		0.000
**B300 – TiO**_2_ **NPs**	0.000	0.000	0.000	0.000	

### Histopathological examination of fetal kidney

**Figure 10:**
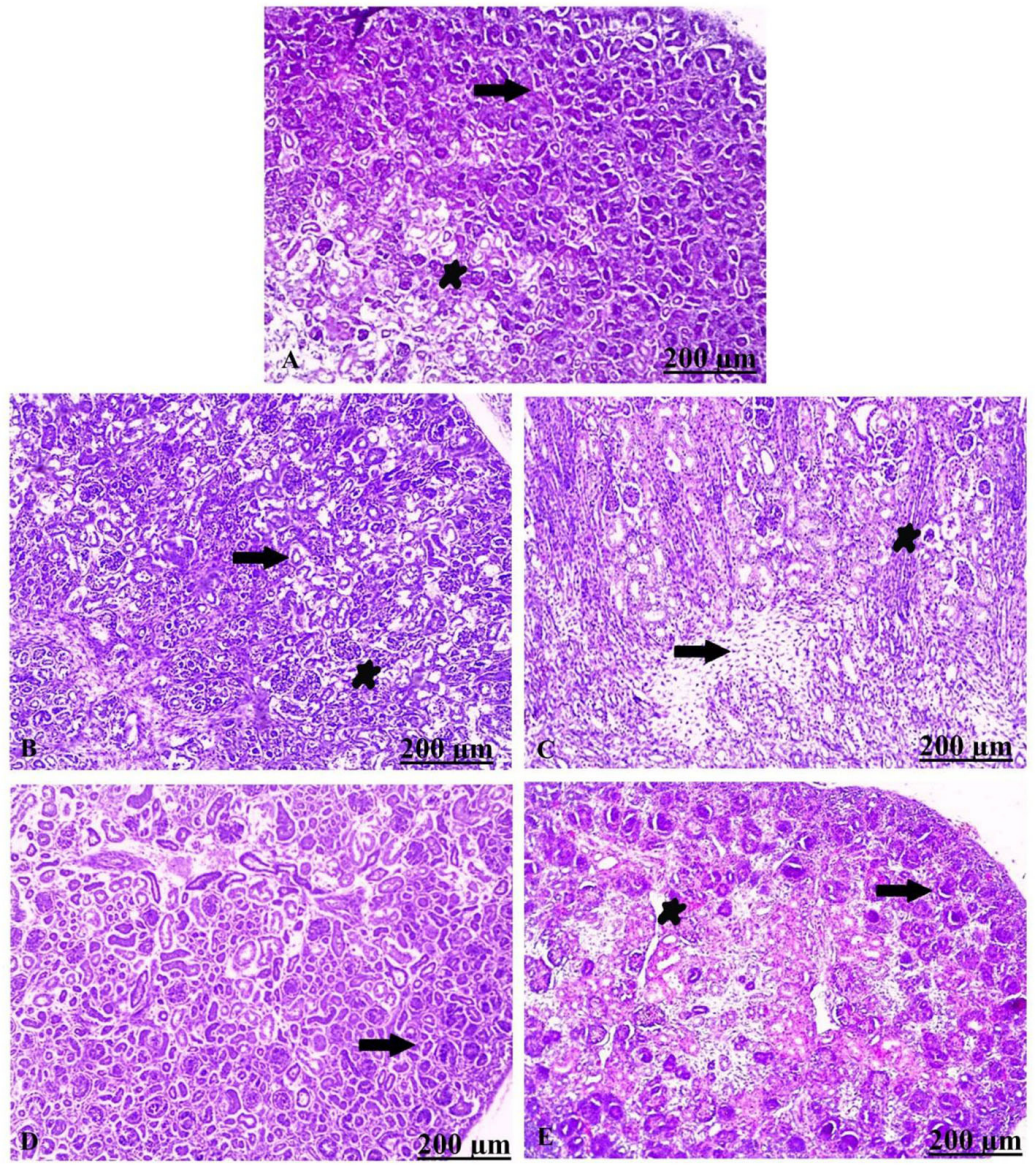
**(A–E)** Transverse sections stained with H&E from fetal kidney of control (untreated) and experimental (treated) groups: **(A)** Light section of control showing intact nephritic tubules (arrow) and glomerulus (star). **(B)** Light section of C100–TiO_2_ NPs showing separation of epithelium-lining tubules from the basement membrane (arrow), besides mononuclear infiltrate (star). **(C)** Light section of C300–TiO_2_ NPs showing necrosis of the renal tubules replaced by inflammatory cells (arrow) as well as desquamated epithelial cells (star). **(D)** Light section of B100–TiO_2_ NPs showing apparently healthy renal parenchyma (arrow). **(E)** Light section of B300–TiO_2_ NPs showing healthy glomeruli (arrow) and mild congestion of the renal blood vessels (star). Scale bar = 200 μm.

Histological findings of kidney tissue stained by H&E exhibited intact renal architectures in the control embryos (Figure 10A). Contrariwise, C100–TiO_2_ NPs mg/kg/day treated group showed necrosis of the renal tissues compensated by focal mononuclear infiltrate and separation of the epithelium-lining tubules (Figure 10B). Similarly, C300–TiO_2_ NPs treated group displayed severe histological damage characterized by necrosed and desquamated epithelial cells with extensive inflammation (Figure 10C). Regarding B100–TiO_2_ NPs, they showed apparently normal kidney parenchyma (Figure 10D). Moreover, treated group with B300–TiO_2_ NPs revealed healthy renal tissues with mildly congested blood vessels (Figure 10E).

## Discussion

Oral consumption of TiO_2_ NPs is considered one of the most common exposure scenarios due to exposure to TiO_2_ NPs found in food, liquid products, and medicines (20, 46). Due to the smaller size and physiochemical properties of TiO_2_ NPs, different cytotoxic and genotoxic behaviors were detected. Previous studies have demonstrated that NPs might pass biological barriers. Following intravenous injection at GD 16–17, TiO_2_ NPs (35 nm) and silica (70 nm) NPs passed the mature blood placenta barrier (59).

Furthermore, another study observed that orally administrated ZrO_2_ might pass the intestinal and maternal blood placenta barriers, and nanoparticles would accumulate in the fetal brain after three repeated oral doses to late-pregnancy mice (GD 16, 17, and 18) (60).

In most multicellular organisms, the mitochondrial DNA is maternally inherited, meaning it is inherited from the mother (61). *16S rRNA* gene was primarily used for the identification of an organism, and thereafter, *16S rRNA* sequencing was able to reclassify the organism into completely new species or even genera (62).

When compared with GC, the entire *16S rRNA* gene exhibits AT richness (63). This was in coordination with our results, where the region amplified by the *16S rRNA* gene was AT-rich.

Based on the results of *16S rRNA* sequences of mothers, TiO_2_ NPs caused genetic variation, where the TiO_2_ NPs-treated groups were genetically distant from the control group that attributed to the effect of TiO_2_ NPs. Landsiedel et al. (64) describe various studies on the genotoxicity of nanomaterials that contain TiO_2_ NPs. They claim that the development of micronuclei, a sign of chromosomal and DNA damage, is evidence of the genotoxicity of TiO_2_ NPs.

DNA damage can result from NPs that enter the body through the skin, mouth, or respiratory system. The oxidative stress and inflammation response associated with exposure to TiO_2_ NPs can interrupt DNA structure, indirectly causing genetic effects. Being small enough, it can directly interact with DNA, causing genetic changes or even damaging the genetic material. When the nuclear membrane vanishes during mitosis, the entry of TiO_2_ NPs into the nucleus is possible. The penetration of TiO_2_ NPs and silica NPs into the nucleus was confirmed by many researchers. They reported that these small particles interact with intracellular proteins, causing aggregation, which can inhibit the replication, transcription, and proliferation processes (65, 66).

Some NPs can enter cell nuclei and may directly interfere with the structure and function of genomic DNA (67). TiO_2_ NPs have been studied for their potential to cause cancer using assays that monitor gene mutations, chromosomal damage, indicative of potential clastogenic activity of the particles, and DNA strand breaks (29, 68–73). In the same context, TiO_2_ NPs cause clastogenicity, genetic variation, oxidative DNA damage, and inflammation *in vivo* in mice. These outcomes were seen after just 5 days of water-based therapy (74).

On the contrary, the results of *16S rRNA* sequences in embryos did not display differences between the control group and the TiO_2_ NP-treated ones, which reflected that the TiO_2_ NPs did not affect the genetic structure of the embryos. This is in coordination with the previous report (30) which reported that at doses up to 1000 mg/kg/day, there was no evidence of toxicity in the maternal or developmental tissues.

On the other hand, the histopathological findings showed that the embryonic renal tissue of the CTiO_2_ NP-treated group showed significant necrosis compensated by focal mononuclear infiltrate, in addition to the separation of epithelium-lining tubules (75). The results indicate that exposure to BTiO_2_ NPs reduced damage in the kidney tissue of the embryo. These results provide evidence that the biosynthesis of NPs can modify their surface due to the interaction with biomolecules, which, in turn, enhances the biocompatibility of the formed NPs.

The results of this study will provide worthy information on the developmental genotoxicity of CTiO_2_ NPs and BTiO_2_ NPs *via* repeated oral exposure, which can help in the process of hazard estimation of widely used nanoparticles.

## Conclusion

Titanium dioxide NPs were biosynthesized with garlic extract as a reducing agent and have a semispherical shape. The biosynthesized protein did not show any structural disorder except for the increase in particle size, which, in turn, caused a little decrease in its cytotoxicity against HepG_2_ cells. Moreover, the results of *16S rRNA* sequences of mother groups showed that chemically synthesized TiO_2_ NPs caused genetic variation compared to the control. The results of the maternal study showed that the biosynthesized TiO_2_ NPs were less toxic compared to chemically synthesized TiO_2_ NPs. However, the embryo-treated groups with both chemically and biosynthesized TiO_2_ NPs did not display any differences compared to the control group. Our study was mainly designed for the experimental use of TiO_2_ NPs and not for food or cosmetics; we recommend that the genetic variation be investigated more carefully.

## Data availability statement

The original contributions presented in the study are included in the article/supplementary material, further inquiries can be directed to the corresponding authors.

## Ethics statement

The animal study was reviewed and approved by the Ethics of Animal Experiments Committee of South Valley University, Faculty of Science (Permit Number: 002/9/22).

## Author contributions

ZK and AS: conception of the idea of the manuscript. ZK, AS, AE, IR, FZ, ZF, and MA: conceptualization and methodology. ZK, AS, AE, IR, FZ, ZF, and MA: formal analysis, writing, reviewing, and editing. All authors have substantially contributed to each step of manuscript preparation, study procedure, contributed to the article, and approved the submitted version.
